# Analysis of the Scalability of UWB Indoor Localization Solutions for High User Densities

**DOI:** 10.3390/s18061875

**Published:** 2018-06-07

**Authors:** Matteo Ridolfi, Samuel Van de Velde, Heidi Steendam, Eli De Poorter

**Affiliations:** 1 IDLab, Department of Information Technology, Ghent University—IMEC, 9000 Gent, Belgium; eli.depoorter@ugent.be; 2 Department of Telecommunications and Information Processing, Ghent University, 9000 Gent, Belgium; samuel.vandevelde@.ugent.be (S.V.d.V.); Heidi.Steendam@UGent.be (H.S.)

**Keywords:** UWB, TDoA, TWR, TDMA, ALOHA, scalability, IPS, user density, Decawave DW1000

## Abstract

Radio frequency (RF) technologies are often used to track assets in indoor environments. Among others, ultra-wideband (UWB) has constantly gained interest thanks to its capability to obtain typical errors of 30 cm or lower, making it more accurate than other wireless technologies such as WiFi, which normally can predict the location with several meters accuracy. However, mainly due to technical requirements that are part of the standard, conventional medium access strategies such as clear channel assessment, are not straightforward to implement. Since most scientific papers focus on UWB accuracy improvements of a single user, it is not clear to which extend this limitation and other design choices impact the scalability of UWB indoor positioning systems. We investigated the scalability of indoor localization solutions, to prove that UWB can be used when hundreds of tags are active in the same system. This paper provides mathematical models that calculate the theoretical supported user density for multiple localization approaches, namely Time Difference of Arrival (TDoA) and Two-Way Ranging (TWR) with different MAC protocol combinations, i.e., ALOHA and TDMA. Moreover, this paper applies these formulas to a number of realistic UWB configurations to study the impact of different UWB schemes and settings. When applied to the 802.15.4a compliant Decawave DW1000 chip, the scalability dramatically degrades if the system operates with uncoordinated protocols and two-way communication schemes. In the best case scenario, UWB DW1000 chips can actively support up to 6171 tags in a single domain cell (no handover) with well-selected settings and choices, i.e., when adopting the combination of TDoA (one-way link) and TDMA. As a consequence, UWB can be used to simultaneously localize thousands of nodes in a dense network. However, we also show that the number of supported devices varies greatly depending on the MAC and PHY configuration choices.

## 1. Introduction

Ultra-wideband (UWB) radios are very popular for accurate indoor localization. Their main unique technology feature is the use of a large frequency band (≥500 MHz) that utilizes trains of pulses with very short duration (less than 1 ns). UWB radios are able to precisely differentiate between pulses that are reflected from different objects. As a result, this technology is very robust to multipath fading, which is crucial in challenging situations such as indoor localization. This timing resolution capability is used for accurate range measurements when UWB is deployed in a Real-Time Locating System (RTLS). Today UWB radio prices are dropping and as such, they are becoming more affordable to the broad market. This explains the great interest in the subject by the scientific community, resulting in a large quantity of recent scientific papers [1,2,3] focusing on UWB indoor localization solutions.

UWB has still some open issues prior to becoming a successful indoor localization choice. The standard imposes certain physical layer (PHY) guidelines such as transmitting power, which may impact the scalability of the system while making it robust against interference with other technologies. Although many scientific papers about UWB localization are available, most of these focus on improving the accuracy of the localization system, without considering scalability aspects. Current UWB localization solutions from scientific literature are typically designed for localizing at most tens of mobile devices (tags) and are evaluated in a small (office) environment or open space area. For example, studies in [2] and [4] demonstrate the high accuracies that can be obtained using UWB (respectively 11 cm and 3.25 mm without post processing), but they are limited to localizing one or two tags in a small area i.e., an office room. Some commercial solutions became recently available and they advertised high update rates but do not mention the conditions in which it is possible. To analyze the scalability of UWB, this paper provides a regulatory overview, an analysis of the impact of design choices (PHY, MAC and localization approach) that can be made for UWB system design and the mathematical framework to calculate the user density based on these choices. Furthermore, to validate the models, we applied them to realistic UWB deployment scenarios.

The remainder of the paper is organized as follows. Section 2 discusses related work. Afterwards Section 3 introduces UWB standard regulations and PHY considerations while, Section 4 focuses on MAC layer aspects as well as the two considered localization techniques, namely TDoA and TWR. Next, Section 5 introduces the scalability models to evaluate the tag density. Finally, in Section 6 the model is applied using realistic settings and schemes of a commercially available UWB chip, followed by the conclusions.

## 2. Related Work

### 2.1. Scientific Literature

UWB is a very well known technology in the scientific community. Originally designed for military communication in the late 1960s [5], it rapidly became popular for other application domains. Its peculiar characteristics allow for precise timing resolution, which is crucial to accurately estimate the Time of Flight (ToF) of the radio signal. Consequently, many researchers started to investigate this promising technology and its relevance in indoor positioning system. Immediately, important studies were published, showing that UWB was capable of localizing a node with a typical error of around 30 cm. Centimeter level accuracy is a significant performance improvement compared to other technologies such as WiFi and Bluetooth low energy (BLE), which can typically achieve localization accuracy in the order of a few meters [6]. The list of studies aiming to improve UWB accuracy is existing and they focus on small-scale setups with a limited number of mobile nodes in a controlled environment such as office or residential rooms. In contrast, very little research has been published that focuses on the scalability of UWB systems. An analysis of the performance of two standard MAC protocol is presented in [7]. It is shown how the overall performance is affected by frame sizes, packet arrival rates and acquisition time. MAC protocol such as the carrier-sense multiple access with collision avoidance (CSMA/CA) protocol in a UWB have weaker performance than in narrowband system due to longer channel acquisition time. To support large numbers of simultaneously transmitting devices, several UWB TDMA solutions have recently been proposed. An overview of these techniques is presented in [8]. In addition, the combination of TDMA with other approaches such as Code Division Multiple Access (CDMA) is investigated in [9], wherein, despite the MAC analysis in terms of latency, it is not shown whether this approach could further enhance the scalability to support higher user densities. A distributed localization scheme is proposed in [10]. The maximum number of users involved in the simulation is 200. The study focuses on collaborative localization based on power measurements (RSSI). However, the impact of their design choices on the overall scalability is not discussed. In [11], an UWB positioning system that supports up to 30 UWB tags in a TDMA access scheme has been developed, but their system is not designed for allowing larger user densities and they only investigate the use of TDoA approaches. A study of the benefits of a hybrid TDoA-TWR approach is illustrated in [12]. In particular, a hybrid algorithm was simulated and afterwards the results were compared with a standard TDoA approach. The experimental results show that in terms of localization precision, the TDoA-TWR approach performs better than the standard TDoA one, but this analysis does not involve any MAC feature nor the study of the system scalability. Thus, it is not possible to evaluate the impact of this solution on the number of supported users. Also in most practical evaluations of commercial systems, the user density is not considered at all. For example, in [13], the authors compare two existing solutions in terms of accuracy, position update rates and end-to-end delays only.

In [14], a system architecture that scales towards more users and has larger coverage areas was proposed. In this architecture, the managing and control part of the network (including allocating UWB resources such as time slots and device roaming) are separated from the actual positioning system. UWB is only used for ranging and position estimation, whereas the other functions are managed through a second non-interfering WiFi network. Nonetheless, coupling communication and localization requirements using UWB only, is also feasible. A possible approach is explained in [15] wherein a contention-based MAC protocol is adapted to gather position information. However, again the scalability of the system in terms of user density is not evaluated.

### 2.2. Scalability of Commercial Solutions

Many UWB companies emerged in the last couple of years, which is another sign of growing attention in UWB localization. While most marketing attention is given to high achievable accuracies, some products also claim to have high scalability with thousands of tags per system. Among different chip manufacturers, DW1000 (Decawave Ltd, Dublin, Ireland) is probably the most used UWB chip. An example of a company that produces UWB devices based on DW1000 is OpenRTLS [16]. On their web page, they claim a maximum update rate of 8000 updates/sec/channel, but also state that the system can accurately localize max 7500 items per second in a 20 m radius. It is not mentioned which settings, localization mechanism and MAC protocol are used to achieve these numbers, nor can these claims be validated.A competitor called Sewio [17] commercializes an UWB TDoA system in which they advertise support for more than 1000 tags. The communication range is around 25–30 m and a table that reports the channel utilization is provided for different packet sizes and refresh rate, e.g., from 0.1 Hz up to 10 Hz. However, there is no information on how high user density scenario are supported and which protocols they implement. Decawave is not the only company that is investing in UWB microchip. Other products are also available in the market. An example is BeSpoon [18], which has developed an UWB microchip and implemented UWB indoor localization systems that support TWR, TDoA and AoA. Similar to previous examples, the figures of user density are somehow vague and there is no quantified support for their claims. In this case, an example of a classic office building with 250 employees is shown. Again, details on adopted techniques are left out of the description. Another commercial UWB chip alternative is Time Domain [19]. In contrast to most commercial chips, Time Domain provides proprietary MAC implementations such as ALOHA and TDMA. However, although the Time Domain datasheet describes the data throughput and expected range and accuracy in a wide range of conditions, the scalability and update rate of the system in each of these conditions is not available. A study that involves multiple Time Domain nodes is available [20]. In an industrial warehouse environment, 22 tags are used to create a low-complexity UWB-based collision avoidance system. Although range estimates are sufficiently accurate to identify dangerous situations, scalability of such a system is not investigated and the authors did not mention how multiple access is implemented, i.e., a suitable MAC protocol.

An overview of the most significant studies and commercial solutions is reported in Table 1. This paper quantifies the scalability claims of the above systems. To this end, mathematical models for the overall scalability are derived that take into account the impact of design aspects such as PHY settings, MAC approach and positioning approaches.

## 3. UWB Regulations and PHY Considerations

A typical RF system aim to serve as many users as possible. Although many techniques exist, they are not always easy to apply and not suited for all kind of technologies. UWB follows strict rules that may limit the applicability of certain mechanisms. The next two paragraphs analyze the PHY layer related aspects that influence the scalability of the system. Similar analysis for MAC and localization schemes is done in Section 4. UWB uses 500 MHz (or 900 MHz) bandwidth and as such could interfere with others operating in the same frequency bands. This is the reason the Federal Communications Commission (FCC) introduced limitations for the maximum UWB radiated power. The harmonized European standard “ETSI EN 302 065-1 Part 2” describes the requirements for UWB location tracking devices. UWB equipment is categorized based on the operational frequency range and the type of tracking it performs. Three different systems are defined:

• LT1 Systems

They operate in the 6 GHz to 9 GHz region of the spectrum and are intended for general localization of people and objects. Devices in this category can be mobile (indoors and outdoors) or fixed (indoors only).

• LT2 Systems

In this category the frequency range is 3.1 GHz to 4.8 GHz and devices are used for person and object tracking and industrial applications. The devices can transmit indoor and outdoor and may be fixed or mobile.

• LAES Systems

Similarly to the previous category, the frequency range is the same 3.1 GHz to 4.8 GHz but in LAES systems, UWB devices are used for tracking staff of emergency services such as firefighters. This implies that these systems are typically temporary and may required licenses prior usage. 

An UWB device can have more than one operating bandwidth provided that the maximum mean power spectral density and the maximum peak power are not above the limits. The maximum mean power spectral density and maximum peak power depend on the type of the UWB system, the frequency and sometimes whether the device is operating outdoor or indoor. All the different values are reported in Table 2 and Table 3 (if not specified, outdoor and indoor are the same). The maximum peak power is specified as the effective isotropic radiated power (e.i.r.p.) contained within a 50 MHz bandwidth at the frequency at which the highest mean radiated power occurs. As noted in the tables, mitigation techniques exist. These allow devices to modulate their transmission power to avoid collisions and do not cause interference with other devices. In general, there are two main categories, namely active and passive mitigation techniques. Each of these includes several mechanisms but only two are applicable to our scenarios, i.e., LT1, LT2 and LEAS. The mitigation techniques are called Low Duty Cycle (LDC) and Detect And Avoid (DAA). Although they are used to improve the robustness of the communications, they may impact the overall scalability of the system.

### 3.1. Detect And Avoid (DAA)

Detect And Avoid is an active mitigation technique, which means that any decision is made upon measurements from the environment. More precisely, it is based on the identification of other radio signals and, if any transmitting device (victim system) is sensed, the TX power is lowered down to a non-interfering level or alternatively, the transmission is deferred until the victim disappears. As outlined in Table 2 and Table 3, a maximum mean e.i.r.p density of −41.3 dBm/MHz and a maximum peak e.i.r.p. of 0 dBm are allowed in the 3.1 GHz–4.8 GHz and 8.5 GHz–9 GHz bands when using DAA but the main criteria to determine which power level can be used, is the distance to the victim system. DAA adopts a zone model wherein different distances correspond to different zones. The main principle is that by sensing the channel it is possible to estimate the distance to the victim system by measuring the power level. The area around the victim is divided in discrete zones and the transmission power is modulated accordingly, e.g., in the last zone, the device can operate without restriction. In a basic model with two zones, i.e., non interference zone and free zone, the signal received from the victim system is compared with a threshold level

(1) $$D_{thresh}=P_{TX\_victim}-I$$

where $P_{TX\_victim}$ is the transmit power of the victim system and *I* is the minimum needed isolation.

Another possibility to mitigate the effect of interfering systems, is to use passive mitigation techniques such as Low Duty Cycle, which is described in the next paragraph.

### 3.2. Low Duty Cycle (LDC)

In this case, no feedback nor measurements from the environment are available. Thus, a passive mitigation technique must use other sources of knowledge. More precisely, LDC limits the activity of the transmitter. This is formally defined by:

(2) $$DC=\frac{T_{on}}{T_{on}+T_{off}}$$

where DC is the duty cycle, $T_{on}$ and $T_{off}$ are the times when the transmitter is on and off, respectively. It is worth mentioning that the timings refer to the entire duration of the UWB pulse frame regardless the pulse repetition frequency (PRF). Besides the limitation imposed by a low duty cycle, i.e., reducing the time spent in transmission by a device, a positive side effect is that a device might be able to increase the transmitted power as it will spend less time in transmission. While low duty cycle could limit the number of transmission per time interval (superframe), the power related aspect can increase the possible coverage of the system and therefore scale it to bigger areas. The LDC restrictions are defined in ECC/DEC/(06)/04, EC DEC 2017/131/EC and EC DEC 2009/343/EC regulations and are listed in Table 4. However, devices belonging to different location tracking categories, have diverse requirements. LT1 systems do not use LDC techniques. LT2 equipment uses a $DC_{max}=5\%$ per transmitter per second and a $T_{on\_max}=25$ ms in the band 3.8 GHz–4.8 GHz by default. Moreover, in the same band and for fixed indoor devices, an additional requirement of 1.5% per minute is mandatory (or an alternative mitigation technique that guarantees the same protection). To conclude, LDC is mandatory requirement in the 3.4 GHz–4.8 GHz band for LAES apparatus.

## 4. Localization Schemes and MAC Considerations

This paragraph discusses general aspects of two ToF-based localization techniques that most often are used in current UWB localization research, namely TDoA and TWR. For each positioning approach, the benefits and drawbacks are outlined.

### 4.1. Time Difference of Arrival

**Figure 1:**
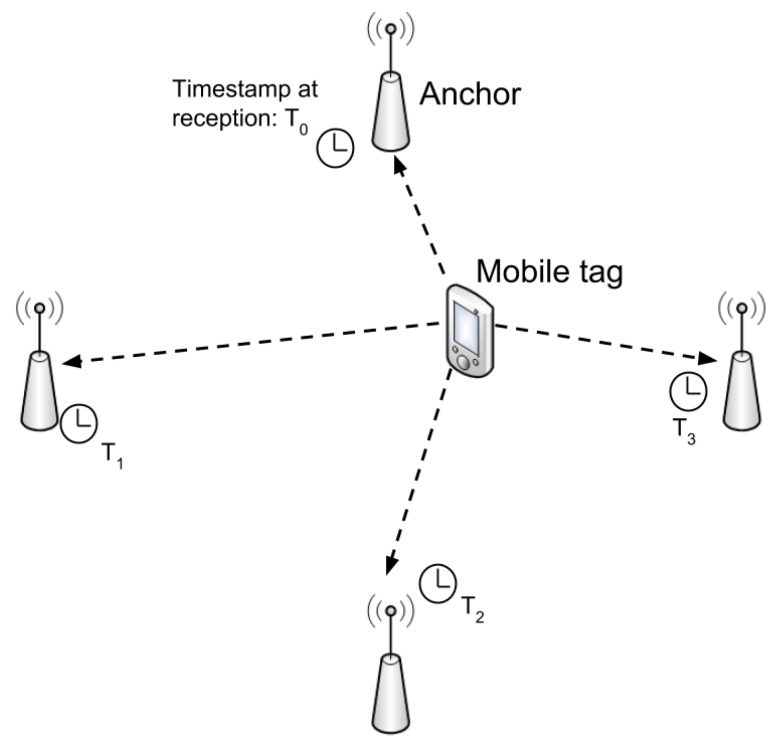
Time Difference of Arrival Scheme.

In TDoA-based location, the mobile tag sends a message that is received by all the anchors in its vicinity. The exact arrival times at each anchor must be communicated to a server, which computes the most likely tag location based on the differences in arrival times. Figure 1 shows the working principle scheme.

The tag blinks at a certain point in time (T_0_) and the message is received by four anchor points at different times (T_1_...T_4_). To estimate the position of the tag, the differences among the arrival times have to be computed [21]. This can be done by the server or alternatively by one of the anchors. For this approach to work, the anchors have to be properly synchronized. Since a 1 ns timing error translates in 30 cm inaccuracy, it is necessary to obtain sub-nanosecond synchronization. This can be achieved by means of expensive hardware such as Rubidium RbXO clocks or alternative implementations as described in [22]. A feasible solution is to use UWB to synchronize the anchors too. This is proven in [23], wherein a comparison of different wireless synchronization algorithms is also presented. In summary, TDoA has the following advantages and disadvantages. 

**Advantages**

- Tags need to send only one packet per position estimate, decreasing energy consumption.
- The server has more computational power than the infrastructure/mobile nodes, resulting in faster position estimation.
- All anchors in range can be used for positioning, so we obtain more accurate results.

**Disadvantages**

- Anchors must be synchronized accurately, which causes synchronization beacons overhead.
- The calculated positions are unknown to the tag, so the estimated position must be communicated via a second network.
- Since the mobile tags are only transmitting and not receiving, data aggregation or collaborative localization is not possible.

### 4.2. Two-Way Ranging

**Figure 2:**
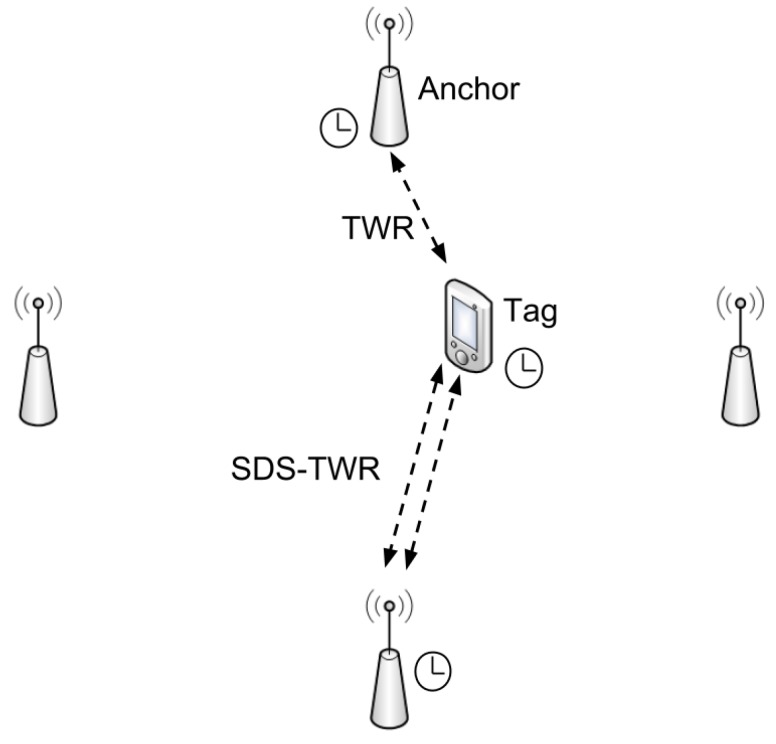
Two Way ranging Scheme.

As the name suggests, a two-way ranging technique requires bidirectional communication between the tag and each of the involved anchors. Two of the most common TWR schemes, called single sided TWR and symmetrical double sided TWR (SDS-TWR), are shown in Figure 2. Generally, the tag initiates the communication sending the first message at time T_0_. The anchor, after a certain period (T_reply_), replies with a message containing the packet timestamps T_1_ and T_2_, which are the reception moments of the first packet and the reply to the tag, respectively. Moreover, these timestamps are unknown to the mobile node. At this point the tag can compute the ToF, which is in turn used to estimate the distance between the tag and the anchor (assuming that the propagation speed is known). A more accurate range estimation is possible if the number of exchanged packets is doubled, e.g., as it happens in SDS-TWR.

The exchanged messages, two or four depending on the TWR algorithm, are used to determine the distances between the anchors and the tag, which, based on the performed distance measurements, will compute its position using one of the previously mentioned localization estimation techniques. This approach also has several advantages and disadvantages.

**Advantages**

- Location could be computed by the mobile nodes.
- Anchors do not have to be synchronized.

**Disadvantages**

- More energy consumption.
- More complex implementation, e.g., selecting the best anchors set, roaming management.
- More messages required for localization.

Table 5 summarizes and compares the two approaches in terms of anchors synchronization, energy overhead, MAC protocol flexibility and estimated position availability.

### 4.3. MAC Considerations

Besides the PHY layer, the medium access control (MAC) layer strongly impacts the scalability of the system. The MAC layer is in charge of properly assigning the medium resources to the competing nodes of the system and as such it impacts among others the overall throughput, the channel access latencies and the overall scalability of the system [24]. Two MAC protocol approaches will be considered in this paper: (i) a random access method where every node competes to gain channel control and (ii) a scheduled access method where the control over the network is granted by a central unit. Regardless the MAC and localization approaches, a significant influence on users scalability is due to frame size. As a rule of thumb, the longer it takes to transmit a packet, the less tags can be localized as the channel is kept busy for a longer period by each transmitting user. The packet duration $T_{packet}$ is standard dependent and its computation follows the standard frame definition. The IEEE 802.15.4-2011 [25] standard determines the frame duration $T_{packet}$ as follows:

(3) $$T_{packet}=T_{SHR}+T_{PHR}+T_{DATA}$$

where:

(4) $$T_{SHR}=(L_{preamble}+L_{SFD})\cdot \tau_{SHR}$$

(5) $$L_{SFD}=\left\{\begin{array}{c} 64\;\mathrm{if}\;\mathrm{bitrate}=110\;\mathrm{kbps}\\ 8\;\;\mathrm{otherwise} \end{array}\right.$$

(6) $$T_{PHR}=21\cdot \tau_{PHR}$$

(7) $$T_{DATA}=(L_{DATA}+L_{Reed})\cdot \tau_{DATA}$$

and the definition of the different variables in (3)–(7) can be found in Table 6.

#### 4.3.1. ALOHA

A typical random access method is ALOHA. This protocol requires no sensing actions to be performed by the nodes before transmitting a packet, i.e., no transmission schedule is necessary. Although simple and straightforward to implement, ALOHA can result in collisions, thereby impacting the scalability of the overall system. In pure ALOHA or simply ALOHA, a transmitting station does not check whether the channel is free or not before transmitting its packet: whenever it has data to send, it transmits. In this scenario the success is only determined by the collision probability with other transmitting nodes. Following the existing scientific literature [26], the maximum channel utilization when using ALOHA is equal to 0,186 which means that only 18.6% of the time can be used for successful, collision free communications. Below this value, 97% of the transmissions are likely to succeed without collisions [27]. This channel utilization can be considered as the capacity of the random access channel. The maximum number of mobile tags with update frequency λ, supported in the system is [26]:

(8) $$N={\left(2\cdot e\cdot T_{packet}\cdot \lambda \right)}^{-1}$$

λ∈[fmin,fmax]

where $T_{packet}$ = transmission duration and *e* = Euler’s number.

More nodes can be added to the network but this would quickly deteriorate the performance of the protocol as shown in [26].

Independently from the localization scheme, ALOHA remains the same. However, when TWR is adopted, we assume that within one frame, two messages are consecutively exchanged between the tag and the anchor in line with the TWR scheme previously described in Section 4.2. As a result, the ALOHA protocol becomes problematic especially when dealing with long messages ($T_{packet}$). The resulting total transmission time $T_{total-TX}$ is computed as follows:

(9) $$T_{total-TX}=n_{anchors}\cdot \left[n_{exchange}\cdot \left(T_{tx}+T_{guard}\right)+\left(n_{exchange}-1\right)\cdot T_{reply}\right]$$

where *n*_*exchange*_ is the number of exchanged messages, e.g., 2 in the TWR case and 4 for SDS-TWR scheme and $n_{anchors}$ is the number of anchors involved in the positioning process, i.e., 4 in a single cell system.

#### 4.3.2. TDMA

**Figure 3:**
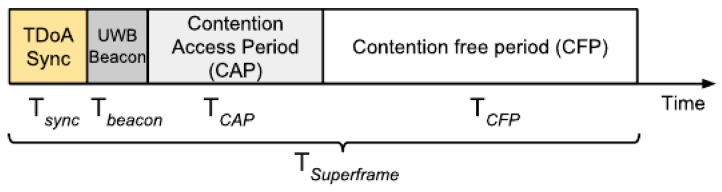
Time Division Multiple Access (TDMA) time division scheme.

In scheduled access approaches, nodes are not allowed to transmit unless they have received a medium resource, which is, for example, a time slot in the TDMA case or a frequency band when using FDMA schemes. Combining multiple of these MAC protocols is also possible and could result in larger supported users at the cost of an increased complexity. The main difference of a TDMA scheme with respect to a random access protocol like ALOHA, is that the transmission of every node is not random but based on scheduled access. Only the user who has gained the right to transmit can initiates the communication. In this way, collisions should be avoided. The most general TDMA approach is to divide the time in superframes, which in turn consist of different parts as shown in Figure 3. This figures summarizes information from multiple section of the ETSI EN 302 065 standard.

The first slot (TDoA Sync) is used to synchronized the anchors nodes and therefore only present when adopting TDoA. The infrastructure nodes are also responsible to sequentially send UWB beacons to inform the mobile tags about the following slot schedule and to indicate the beginning of the contention access period (CAP). When a new tag joins the network, it requests a time slot during CAP using a random MAC protocol such as ALOHA. Once the scheduler assigns resources to a tag, the latter can communicate with the anchors without worrying about collisions with other nodes. This happens during the contention free period (CFP), which size is computed as follows.

(10) $$T_{CFP}=n_{resources}\cdot T_{packet}$$

Consequently, to evaluate the maximum number of tags that can be accommodated with TDMA we can use (11).

(11) $$n_{resources}=\frac{T_{Superframe}-T_{CAP}-T_{Sync}-T_{beacon}}{T_{packet}}$$

It is important to remember that using ALOHA during the CAP period, collisions may happen, which means that certain nodes would have to try to send more than one slot request, resulting in delayed access to the network and higher energy consumption.

When TDMA is chosen in a TWR network, as it happens for ALOHA, more packets are exchanged and therefore the the slot size increases according to (9).

#### 4.3.3. Hybrid TDMA/FDMA

Another possibility is to combine multiple techniques. For example, using at the same time TDMA and FDMA, it is possible to have the anchor points working on different non-interfering channels in order to serve simultaneously more users, e.g., the UWB Decawave DW1000 chip can operate in 6 separate channels. Using different preamble codes is also an option to increase diversity.Similarly as in the TDMA-TWR case, the position will be known after ranging with all the four anchors in the cell but the localization process becomes more efficient. This is because multiple users can simultaneously communicate with different anchor points on separate channels without interfering with each other. The gain is proportional to degree of diversity, if the system is using two channels then double $2\times n_{resources}$ will be available.

In the TDoA case, however, a combination of two different MAC schemes such as TDMA and FDMA, is not a feasible solution. If there are four anchors on four different channels and one tag is broadcasting a message in one of these four channels, then the message is received by one anchor only and the position cannot be computed. Assuming a single frequency antenna, it would be necessary to deploy extra hardware to overcome this limitation. If the system has to operate on more than one channel, then each anchor node needs to have more than one device, each of them operating on a separate frequency band as every device can operate in a single frequency band at the same time only.

## 5. Scalability Model

**Figure 4:**
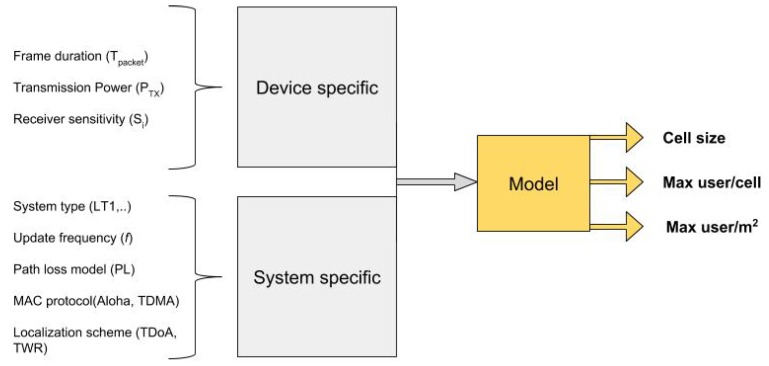
System diagram for UWB indoor scalability model.

In the previous sections, we provided mathematical formulas of the different PHY, MAC and localization approaches. In this section, we combine these formulas to create a full-system scalability model. This is used to derive the maximum user density in an UWB network with the aforementioned characteristic. In Figure 4, an overview of what is taken into account when studying the scalability is outlined. Each input impacts the overall scalability of the UWB localization system. With this framework, we want to simplify the analysis of a network in which as many users as possible can be localized/tracked. Therefore, the steps to compute the maximum user density are kept as general as possible. Some relevant examples are also provided in Section 6 to validate the scalability model using the characteristics of the commercial Decawave DW1000 chip. In the following paragraph, a list of the steps in order to evaluate the achievable user density is presented.

### 5.1. Model Steps

#### 5.1.1. Analyze system requirements

Several aspects influence the analysis and therefore, they have to be defined.

1. Specify the type of system, e.g., LT1, LT2 or LAES. Implications of this are explained in Section 3.
2. Specify the radio channel, e.g., 6 channels are available with DW1000.
3. Decide whether the system is going to be deployed indoor or outdoor. Consequences of this aspect are also shown in Table 2 and Table 3.
4. Upon previous decisions, investigate possible mitigation techniques effects. DAA and LDC are describe in Section 3.1 and Section 3.2, respectively.

#### 5.1.2. Achievable Range

The maximum range that a device can achieve ($d_{MAX}$) is one of the main indicator to study how scalable a solution can be. Known the shape of the cell, e.g., square, circle or hexagonal, it is possible to obtain the area that can be covered for specific UWB settings.

**Figure 5:**
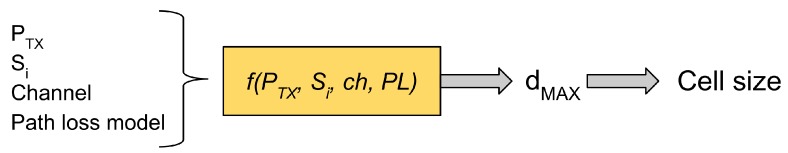
Cell size definition problem.

The formula to compute $d_{MAX}$ is function of the transmission power ($P_{TX}$), receiver sensitivity ($S_{i}$), channel (ch) and mainly varies based on the path loss (PL) model that is adopted to study the system (Figure 5). The output of this step is also an important parameter to evaluate coverage capabilities of UWB.

#### 5.1.3. Allocate System Resources

**Figure 6:**
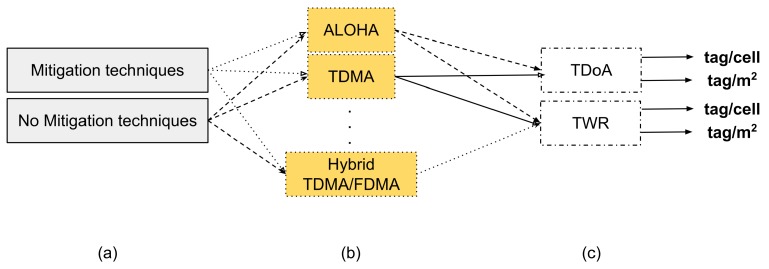
Final step to output the maximum user density achievable (**a**) Standard constraints (**b**) MAC protocol (**c**) localization technique.

The last but the most crucial step concerns the behaviour of the system in assigning the resources to the tags and in estimating their positions. Considering all the system and device variables that play a role in this regard, there are many possible combinations, as can be seen in Figure 6.

The first part (a) is essentially the result of all choices made in the first step, which defines the system type and the operational frequency. Then, one or combination of some MAC protocols are used to assign resources (b). However, only after the third action (c) is possible to estimate the maximum tags supported in the system. The output can be represented both in terms of tags/cell and tag/m^2^.

## 6. Application of the Scalability Model

In this section, we validate the scalability model defined in Section 5 mainly using MATLAB to compare different scenarios. To this end, we refer to UWB properties of the commercial DW1000 chip by Decawave Ltd., which allows to easily adjust operational modes. Since we also want to show the impact of different UWB configurations on user density, we define and use two of them in combination with diverse MAC protocols and localization approaches.

Configuration 1

- System type: LT1
- Channel: 5 (center frequency = 6489.6 MHz and bandwidth = 500 MHz)
- bitrate: 6.81 Mbps
- Preamble: 128 symbols
- PRF: 16 MHz
- Payload: 3 bytes
- Number of anchors: 4

Configuration related variables: $T_{packet-conf1}$ = packet duration, $S_{i-conf1}$ = receiver sensitivity, $d_{MAX-conf1}$ = maximum theoretical range.

Configuration 2

- System type: LT1
- Channel: 5
- bitrate: 110 kbps
- Preamble: 4096 symbols
- PRF: 64 MHz
- Payload: 3 bytes
- Number of anchors: 4

Configuration related variables: $T_{packet-conf2}$ = packet duration, $S_{i-conf2}$ = receiver sensitivity, $d_{MAX-conf2}$ = maximum theoretical range.

The first step requires to have a full understanding of the system. According to standard definitions [25] and UWB regulations (see Section 3), we assume that both configurations are LT1 type operating in channel 5. Moreover, considering that localization is done indoor, the two systems do not have to use any mitigation technique.

To compute the frame duration for each scenario, we have to introduce the Decawave symbol duration table, which reports all the timings depending on the frame part. Using the values of Table 7 and (3) we find the frame duration for configuration 1 and 2, which are $T_{packet-conf1}=162\;\mu s$ and $T_{packet-conf2}=4.7$ ms.

Before using these values in the scalability model, we want to calculate the achievable range, which is the second step of the scalability model described in Section 5.1 and in turns defines the maximum cell size. As shown in Figure 5, we still need to define the transmission power, the receiver sensitivity and the path loss model. In this case, the latter is the Free Space path loss (FSPL), which is derived from Friis Transmission formula [28]. The equation to describe the attenuation in dB is the following:

(12) FSPL(dB)=32.45+20log(d[km])+20log(f[MHz])

As we also assume to have 0 dBi for the antenna gain and no other attenuation, (12) can be used to obtain the $d_{MAX}$. To quantify the maximum range, it is necessary to link the transmission power $P_{TX}$ and the receiver sensitivity $S_{i}$, which can be found in the Decawave manual and it corresponds to $S_{i-conf1}=-93$ dBm and $S_{i-conf2}=-106$ dBm. Together with $P_{TX}=-14.3$ dBm, this results in available link margin of 82 dB (configuration 1) and 92 dB (configuration 2). Therefore, from (12) and using the available link budget, we find that $d_{MAX-conf1}=46$ m and $d_{MAX-conf2}=146$ m.

**Figure 7:**
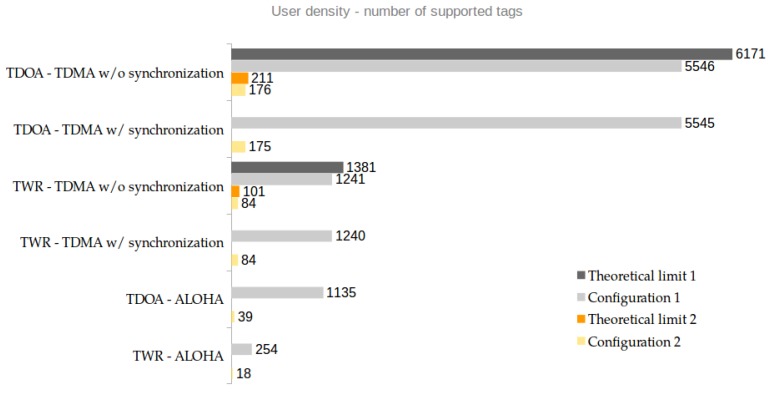
Tag density computed applying the scalability model with two different UWB configurations and two extra reference modes for TDMA w/o synchronization.

Finally, the last step of the model takes all aspects into account (Figure 6) and depending on localization scheme and MAC protocol, it outputs the maximum user densities, as shown in Figure 7. In Section 4.3, we explained how the available resources per tag are computed and for that we also defined other variable, which are common to both configurations and here listed:

- $T_{reply}=400\;\mu s$
- $T_{superframe}=1$ s
- $T_{CAP}=100$ ms
- $T_{synch}=0$ (no synch) or $T_{synch}=T_{packet}$
- $T_{beacon}=T_{packet}$ with max payload (1023 bytes)

An extra mode, called "theoretical limit" is also shown in Figure 7 for the TDoA w/o synchronization case. The purpose is to show the upper theoretical limit, in which no contention access period is present since all the slot contention and assignations are done at another level, e.g., using a backbone network. This also reduces the $T_{beacon}$ since no scheduling has to be distributed among the tags, i.e., $T_{beacon}=T_{packet}$.

The main outcome is that the settings that determine the duration of the packet are very important when design an UWB indoor localization system. It is clearly shown that a short packet, i.e., configuration 1, outperforms a longer one in terms of tag density. In TDoA-TDMA, when adopting short packet option, the system is capable of supporting up to 6171 updates per second in the limit case, while using the longer 4.7 ms packet, only 211 tags are supported, which is around 97% less than in theoretical limit 1 case. Moreover, the localization scheme has also a big influence on the system scalability. When working with the same MAC approach, i.e., TDMA, the difference in tag density between TDoA and TWR is significant. The latter can only support 22% of the maximum number of tags in the short packet theoretical limit mode. For reference configuration 2, TWR-TDMA supports 53% less tags than the correspondent TDoA-TDMA case. As each application scenario has its own requirements in terms of tag density, a random access approach (ALOHA) combined with short TDOA packets can still be considered scalable, i.e., 1135 supported updates/s are probably enough to guarantee access in a single-floor scenario with a few offices. On the other hand, TWR-ALOHA has a very limited number of updates/s, both in configuration 1 and 2. The only advantage of this configuration is that ALOHA is easy to implement on the mobile tags and the infrastructure nodes do not need synchronization. Therefore, when only a few tags need to be localized at low update frequency this working setup can be used. The scalability model allows for further analysis that can be useful in various applications.

**Figure 8:**
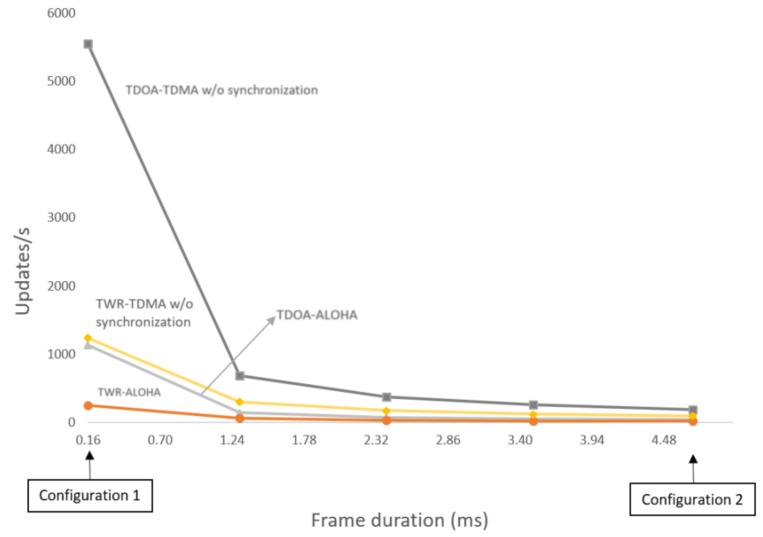
Tag density in relation to frame length and applied to four different configurations.

In Figure 8, the number of supported updates/s is plotted against the frame duration. Between the two configurations used in this Section, more frame lengths have been used. Afterwards, the study has been applied to different UWB schemes, i.e., TDOA-TDMA, TWR-TDMA, TDOA-ALOHA and TWR-ALOHA. The graph clearly shows a non linear behaviour as the tag density drops dramatically for frame lengths bigger than 0.5 ms and then decreases slowly to the minimum, which in this case corresponds to configuration 2. To obtain this graph, we applied formulas of the scalability models that are described in Section 4, wherein the different MAC protocols and localization techniques are defined. When designing an UWB system for indoor localization, if the target density is known, a graph like the one in Figure 8, can be used to find which UWB schemes satisfy the requirements. For example, if 1000 is the required tag density, there are mainly three options: TWR-TDMA or TDOA-ALOHA with a frame duration of 0.16 ms or alternatively TDOA-TDMA with around 1.1 ms packet length.

More configuration, custom packets and options can be tested and evaluated using the model provided in this paper. It is possible to study the impact of different solutions, which is very important in a complex structure such as an indoor localization system.

## 7. Conclusions

Ultra-wideband has been showing an enormous potential to be adopted in any indoor localization scenario, it can localize a tag with cm-level accuracy, which is better than traditional RF technologies. However, its main drawback is the limited scalability in terms of tag density due to the lack of collision avoidance capabilities. We gave an overview of the regulations that have been drafted, and showed that the supported tag density can mathematically be calculated and provide the necessary formulas to calculate the impact of different UWB PHY settings, MAC protocol and localization approach. These impacts are combined in a scalability model that takes into account all the possible aspects that could impact the tag density. In three simple steps it is possible to evaluate how many users the system can ultimately support. Moreover, to validate the model, we applied it to an existing commercial solution, which allows to configure various settings. We created two configurations and then compared the outcomes for different MAC-localization scheme scenarios. We showed that with specific setting, i.e., TDoA-TDMA and short packets, UWB is capable of accommodating resources for thousands of users per second, which is a high scalable solution. However, as expected, the results vary with different inputs. The outcomes of this paper provide insights in the trade-offs for UWB full-system design, and allow UWB solution designers to determine a priori the expected number of supported users.

## Figures and Tables

**Table 1:** Related work overview.

Solution	Approach	User Density	Other Notes
Subramanian and Lim, 2005 [10]	RSSI distributed localization	200 tags	Results of simulation
Khun et al.; 2011 [11]	TDoA	30 tags	Real life experiments with TDMA protocol
Kolakpwski and Djaja-Josko, 2016 [12]	Hybrid TDoA-TWR	Not evaluated	Improved system accuracy
Monica and Ferrari, 2016 [20]	TWR	22 tags	Industrial warehouse
OpenRTLS [16]	TDoA, TWR	7500 updates/s	Communication range 20 m
Sewio [17]	TDoA	1000 updates/s	Communication range 30 m
Be Spoon [18]	TDoA, TWR, AoA	750 updates/s	Office like environment
Time Domain [19]	TWR	Unknown	Proprietary MAC protocols

**Table 2:** Maximum mean power spectral density (dBm/MHz) for UWB systems.

Frequency (GHz)	Maximum Mean Power Spectral Density (e.i.r.p.) (dBm/MHz)		
	LT1	LT2	LEAS
f≤1.6	−90	−90	−90
1.6<f≤2.7	−85	−85	−85
2.7<f≤3.1	−70	−70	−70
3.1<f≤3.4	−70	−70 (−41.3 with DAA) ^1^	−70 (−41.3 with DAA) ^1^
3.4<f≤3.8	−80	−41.3 ^1,2^ (see note 1 for fixed outdoor)	−21.3 ^1^
3.8<f≤4.2	−70	−41.3 ^1,2^ (see note 1 and 3 for outdoor)	−21.3 ^1^
4.2<f≤4.8	−70	−41.3 ^1,2^ (see note 1 and 3 for outdoor)	−41.3 ^1^
4.8<f≤6	−70	−70	−70
6<f≤8.5	−41.3	−70	−70
8.5<f≤9	−64 (−41.3 with DAA)	−70	−70
9<f≤10.6	−65	−70	−70
*f* >10.6	−85	−85	−85

^1^ Duty cycle of maximum 5% per second per transmitter on time (Ton). ^2^ Duty cycle limited to 1.5% per minute per transmitter on time (Ton) or an alternative mitigation technique should be used to achieve the same protection. ^3^ The maximum mean e.i.r.p. spectral density in the band 4.2 GHz to 4.4 GHz for emissions that appear 30° or greater above the horizontal plane should be less than −47.3 dBm/MHz, see clause 4.6.1.4 ECC/REC(11)09.

**Table 3:** Maximum peak power (dBm in 50 MHz) for UWB systems.

Frequency (GHz)	Maximum Peak Power Spectral Density (e.i.r.p.) (dBm in 50 MHz)		
	LT1	LT2	LEAS
f≤1.6	−50	−50	−50
1.6<f≤2.7	−45	−45	−45
2.7<f≤3.1	−36	−36	−36
3.1<f≤3.4	−36	−36 (0 with DAA) ^1^	−36 (0 with DAA) ^1^
3.4<f≤3.8	−40	0 ^1,2^ (see note 1 for fixed outdoor)	20 ^1^
3.8<f≤4.2	−60	0 ^1,2^ (see note 1 and 3 for outdoor)	20 ^1^
4.2<f≤4.8	−60	0 ^1,2^ (see note 1 and 3 for outdoor)	0 ^1^
4.8<f≤6	−30	−30	−30
6<f≤8.5	0	−30	−30
8.5<f≤9	−25 (0 with DAA)	−70	−30
9<f≤10.6	−25	−30	−30
*f* > 10.6	−45	−45	−45

^1^ Duty cycle of maximum 5% per second per transmitter on time (Ton). ^2^ Duty cycle limited to 1.5% per minute per transmitter on time (Ton) or an alternative mitigation technique should be used to achieve the same protection. ^3^ The maximum mean e.i.r.p. spectral density in the band 4.2 GHz to 4.4 GHz for emissions that appear 30° or greater above the horizontal plane should be less than −47.3 dBm/MHz, see clause 4.6.1.4 ECC/REC(11)09.

**Table 4:** Low Duty Cycle (LDC) restrictions for UWB technology.

Parameters	Restrictions
Max transmitter on time $T_{on\_max}$	5 ms
Mean transmitter off time $T_{off\_mean}$	≥38 ms (averaged over 1 s)
Sum transmitter off time $\sum T_{off}$	>950 ms per second
Sum transmitter on time $\sum T_{on}$	<18 s per hour

**Table 5:** TDoA and TWR approaches comparison

	Time Difference of Arrival	Two Way Ranging
Anchors Synchronization	Yes	No
Energy Overhead	Low	High
Hybrid MAC Protocol Flexibility	Low	High
Position Information Availability	At Infrastructure Side	At Both Sides

**Table 6:** UWB variables: radio, MAC, standard levels.

	Symbol	Variable Name	Unit
Radio	$T_{packet}$	Frame duration	ms
	$T_{SHR}$	Synchronization header (SHR) duration	ms
	$T_{PHR}$	PHY header (PHR) duration	ms
	$T_{DATA}$	PHY service data unit duration	ms
	$L_{preamble}$	Preamble length	symbols
	$L_{SFD}$	Start of frame delimiter (SFD) length	bits
	$L_{Reed}$	Reed Solomon bits	bits
	$L_{DATA}$	Data length	bits
	$\tau_{SHR}$	SHR symbols duration	ms
	$\tau_{PHR}$	PHR symbols duration	ms
	$\tau_{DATA}$	Data symbols duration	ms
	PRF	Pulse repetition frequency	Hz
	$P_{TX}$	Transmission power	dBm
	$S_{i}$	Receiver sensitivity	dBm
	Ch	Channel number	unitless
	PL	Path loss model	unitless
MAC	$T_{reply}$	Reply time in TWR	ms
	$T_{tx}$	Transmission duration	ms
	$T_{sync}$	Synchronization beacon duration	ms
	$T_{beacon}$	Beacon duration	ms
	$T_{CAP}$	Contention Access Period (CAP) duration	ms
	$T_{CFP}$	Contention Free Period (CFP) duration	ms
	$T_{superframe}$	Superframe duration	ms
	$n_{resources}$	Resources available	unitless
	$n_{anchors}$	Number of anchors	unitless
	$n_{exchange}$	Number of message exchanges	unitless
	*N*	Number of supported tags	unitless
	λ	Position update frequency	Hz
LDC	$T_{on}$	Transmitter on time	ms
	$T_{off}$	Transmitter off time	ms
DAA	$D_{thresh}$	Power level threshold	dBm
	$P_{TX_{victim}}$	Transmit power of victim system	dBm
	*I*	Minimum needed isolation	dBm

**Table 7:** DW1000 Symbols duration.

Data Rate (Mbps)	PRF (MHz)	SHR (ns)	PHR (ns)	Data (ns)
0.11	16	993.59	8205.13	8205.13
0.11	64	1017.63	8205.13	8205.13
0.85	16	993.59	1025.64	1025.64
0.85	64	1017.63	1025.64	1025.64
6.81	16	993.59	1025.64	128.21
6.81	64	1017.63	1025.64	128.21

## Abbreviations

The following abbreviations are used in this manuscript:

RF: Radio frequency

UWB: ultra-wideband

TDoA: Time difference of arrival

TWR: Two-way ranging

RTLS: Real-time locating systems

PHY: Physical layer

ToF: Time of flight

BLE: Bluetooth low energy

CSMA/CA: Carrier-sense multiple access with collision avoidance

CDMA: Code division multiple access

RSSI: Received signal strength indicator

FCC: Federal Communication Commission

e.i.r.p: Effective isotropic radiated power

LDC: Low duty cycle

DAA: Detect and avoid

PRF: Pulse repetition frequency

SDS-TWR: Symmetrical double sided two-way ranging

MAC: Medium access control

SHR: Synchronization header

PHY: Physical layer header

SFD: Start of frame delimiter

CAP: Contention access period

CFP: Contention free period

TDMA: Time division multiple access

PL: Path loss

FSPL: Free space path loss

## References

[1] Guvenc I., Gezici S., Sahinoglu Z. Ultra-wideband range estimation: Theoretical limits and practical algorithms, in Ultra-Wideband. Proceedings of the 2008 IEEE International Conference on Ultra-Wideband (ICUWB).

[2] Silva B., Pang Z., Åkerberg J., Neander J., Hancke G. Experimental Study of UWB-based High Precision Localization for Industrial Applications. Proceedings of the 2014 IEEE International Conference on Ultra-WideBand (ICUWB).

[3] Lo A., Yarovoy A., Bauge T., Russell M., Harmer D., Kull B. (2011). An Ultra-Wideband Ad Hoc Sensor Network for Real Time Indoor Localization of Emergency Responders.

[4] Rowe N.C., Fathy A.E., Kuhn M.J., Mahfouz M.R. A UWB Transmit-Only Based Scheme for Multi-Tag Support in a Millimeter Accuracy Localization. Proceedings of the IEEE Topical Conference on Wireless Sensors and Sensor Networks (WiSNet).

[5] Barrett T.W. History of Ultra-WideBand (UWB) Radar & Communications: Pioneers and Innovators. Proceedings of the Progress in Electromagnetics Symposium.

[6] Koyuncu H., Yang S.H. A survey of indoor positioning and object locating systems. Proceedings of the International Conference on Innovations in Information Technology.

[7] Ding J., Zhao L., Medidi S.R., Sivalingam K.M. MAC protocols for ultra-wide-band (UWB) wireless networks: Impact of channel acquisition time. Proceedings of the SPIE 4869, Emerging Technologies for Future Generation Wireless Communications.

[8] Gupta A., Mohapatra P. (2007). A survey on Ultra Wide Band Medium Access Control Schemes. Comput. Netw..

[9] Janssen M., Busboom A., Schoon U., Koch C., Colln G.V. A Hybrid MAC Layer for Localization and Data. Proceedings of the IEEE 17th Conference on Emerging Technologies and Factory Automation (ETFA).

[10] Subramanian A., Lim J.G. A Scalable UWB Based Scheme for Localization in Wireless Networks. Proceedings of the Conference Record of the Thirty-Ninth Asilomar Conference on Signals, Systems and Computers.

[11] Kuhn M.J., Mahfouz M.R., Turnmire J., Wang Y., Fathy A.E. A Multi-Tag Access Scheme for Indoor UWB Localization Systems used in Medical Environments. Proceedings of the 2011 IEEE Topical Conference on Biomedical Wireless Technologies, Networks, and Sensing Systems (BioWireleSS).

[12] Kolakowski M., Djaja-Josko V. TDoA-TWR based positioning algorithm for UWB localization system. Proceedings of the 2016 21st International Conference on Microwave, Radar and Wireless Communications (MIKON).

[13] Hammer F., Yudanto R., Neumann K., Pichler M., Cockx J., Niestroj C., Petré F. (2016). Performance Evaluation of 3D-Position Estimation Systems. IEEE Sens. J..

[14] Ridolfi M., Van de Velde S., Steendam H., De Poorter E. WiFi Ad-Hoc Mesh Network and MAC Protocol Solution for UWB Indoor Localization System. Proceedings of the 23rd IEEE Symposium on Communications and Vehicular Technology in the Benelux (SCVT).

[15] Alcock P., Roedig U., Hazas M. Combining Positioning and Communication Using UWB Transceivers. Proceedings of the 5th IEEE International Conference, DCOSS 2009.

[16] OpenRTLS. https://openrtls.com/.

[17] sewio. https://www.sewio.net/.

[18] BeSpoon. http://bespoon.com/.

[19] Time Domain. https://timedomain.com/.

[20] Monica S., Ferrari G. (2016). Low-complexity UWB-based collision avoidance system for automated guided vehicles. ICT Express.

[21] Bai Y., Lu X. Research on UWB Indoor Positioning Based on TDoA Technique. Proceedings of the 2009 9th International Conference on Electronic Measurement & Instruments.

[22] Djaja-Josko V., Kolakowski J. A new method for wireless synchronization and TDoA error reduction in UWB positioning system. Proceedings of the 2016 21st International Conference on Microwave, Radar and Wireless Communications (MIKON).

[23] McElroy C., Neirynck D., McLaughlin M. Comparison of wireless clock synchronization algorithms for indoor location systems. Proceedings of the 2014 IEEE International Conference on Communications Workshops (ICC).

[24] De Nardis L., Di Benedetto M.G. (2003). Medium Access Control Design for UWB communication systems. J. Commun. Netw..

[25] IEEE Standard for Local and Metropolitan Area Networks–Part 15.4: Low-Rate Wireless Personal Area Networks (LR-WPANs). https://standards.ieee.org/findstds/standard/802.15.4-2011.html.

[26] Abramson N. The ALOHA system—Another Alternative for Computer Communications. Proceedings of the Fall Joint Computer Conference.

[27] ScenSor DW1000. http://www.decawave.com/products/dw1000.

[28] Buehrer R.M., Davis W.A., Safaai-Jazi A., Sweeney D. Characterization of the ultra-wideband channel. Proceedings of the IEEE Conference on Ultra Wideband Systems and Technologies.

